# Profiles of Structural Violence in Hispanic/Latino Immigrant and Non-Immigrant Parents: the Hispanic Community Health Study/Study of Latinos (HCHS/SOL) Youth Study

**DOI:** 10.1007/s10903-025-01707-9

**Published:** 2025-06-18

**Authors:** Kristine Molina, Kevin Tan, Jinsong Chen, Ramon Durazo-Arvizu, Linda C. Gallo, Krista M. Perreira, Lisa Sanchez-Johnsen, Donglin Zeng, Elizabeth Pulgaron, Alan Delamater, Sage Kim, Paula G. Allen-Meares, Martha L. Daviglus, Carmen R. Isasi, Rosalba Hernandez

**Affiliations:** 1 University of California, Irvine, Irvine, USA; 2 University of Illinois Urbana-Champaign, Urbana, USA; 3 University of Nevada Reno, Reno, USA; 4 University of Illinois Chicago, Chicago, USA; 5 University of Southern California, Los Angeles, USA; 6 San Diego State University, San Diego, USA; 7 University of North Carolina at Chapel Hill, Chapel Hill, USA; 8 Medical College of Wisconsin, Milwaukee, USA; 9 University of Miami, Miami, USA; 10 Northwestern University Feinberg School of Medicine, Chicago, USA; 11 Albert Einstein College of Medicine, Bronx, USA

**Keywords:** Structural violence, Hispanic/latino caregivers, Stress, Socioeconomic disparities, Latent profile analysis, Immigrant status

## Abstract

This study provides critical insights into the pervasive structural violence and compounded stress experienced by Hispanic/Latino caregivers, a population disproportionately impacted by systemic inequities that can adversely affect overall well-being. We analyzed data from the Hispanic Community Health Study/Study of Latinos Youth (*n* = 458) to identify structural violence exposure patterns among Hispanic/Latino caregivers/parents and examine how these patterns vary by birthplace and U.S. residency duration. Structural violence exposure was measured using six domains: chronic stress, perceived stress, racial/ethnic discrimination, acculturative stress, economic hardship, and neighborhood disorder. Latent profile and multinomial logistic regression identified structural violence patterns and their links to birthplace and U.S. residency. Participants were on average 38.3 years (range 23–71), were predominantly female (87.6%), mostly of Mexican descent (45.9%), and had low socioeconomic status, with 32.7% lacking a high school diploma, 50.1% earning under $20,000 annually, and 84.9% foreign-born. Latent profile analyses revealed three distinct exposure patterns: *low-exposure*, *high-exposure*, and *high-environmental exposure*. Findings indicate that foreign-born Hispanics/Latinos with less than 10 years in the U.S. had significantly lower odds of *high-environmental exposure* compared to their U.S.-born counterparts, suggesting differential vulnerability based on immigration status and time in the U.S. Birthplace and years in the U.S. were not significantly associated with group membership when comparing high- and low-exposure groups, indicating that multiple factors beyond nativity may contribute to structural violence experiences. These findings underscore the critical need for context-specific interventions tailored to the unique stressors faced by Hispanic/Latino communities to mitigate health risks associated with structural violence.

## Introduction

Data from the Hispanic Community Health Study/Study of Latinos (HCHS/SOL), the largest U.S. cohort of Hispanics/Latinos, indicates that nearly half of parental caregivers are currently managing one to two chronic stressors, such as economic insecurity, unstable employment, immigration-related challenges, or exposure to community violence—factors that reflect broader systemic inequalities. These stressors are linked to increased health risks in their children [1], underscoring the impact of caregiver stress on child health outcomes. Yet with few exceptions [2–4], most studies on “parental stress” focus primarily on the stress stemming from caregiving responsibilities, often neglecting stressors from the broader ecological context, such as financial strain and daily encounters [5].

Acknowledging the ecological context necessitates addressing societal systems that impede individuals and communities from thriving, largely due to unequal power distribution and resource disparities [6]. This phenomenon, known as structural violence, refers to the macrolevel systems, social forces, and institutions that perpetuate inequities among racial/ethnic groups [7–9]. It includes societal factors such as racism, classism, geographic disparities, and legal violence, all of which contribute to instability and fear, further compounding the stress experienced by individuals and communities.

As a racialized group, U.S. Hispanics/Latinos face structural violence in multiple forms, including restrictive immigration policies, neighborhood disorder, and racial discrimination. These factors heighten race-related vulnerability and exacerbate health disparities. Hispanic/Latino parents, whether U.S.-born or immigrant, report higher levels of parental stress compared to other ethnicities, as well as more frequent encounters with everyday racism than White peers [10]. In a study of immigrant Hispanic/Latino parents, 30% reported significant adverse pre-migration life events, 13% experienced trauma (e.g., assault), and one-third reported post-migration racial discrimination [11]. Moreover, stressors often co-occur, with those reporting racial/ethnic discrimination also noting heightened race-related tension in their neighborhood [11]. Thus, *parental stress* should be understood within the broader social context, emphasizing the need to address social determinants of health and harmful societal structures (Belsky, 1984, as cited by Ornelas and Perreira [11]).

A key gap exists in understanding the ecological context for Hispanic/Latino parents, especially regarding the overlap of structural violence and stress. This group faces unique stressors, such as financial strain, acculturation gaps, and race-related challenges their children encounter, including bullying and anxiety [1, 11–13]. Addressing these challenges is crucial for developing tailored support strategies to improve youth outcomes.

### Complex Stress Patterns Stemming from Structural Violence

Researchers often classify perceived stress, chronic stress, childhood adversities, and traumatic stress as non-racialized. However, structural violence includes stressors that, while not explicitly linked to race/ethnicity, carry racial implications. For instance, financial strain from unemployment or exploitative conditions, caregiving challenges resulting from resource scarcity and limited social safety nets, and trauma from neighborhood violence often intersect with lower societal status, disproportionately impacting Hispanics/Latinos and other racial/ethnic minorities.

Methodological approaches to measuring stress from structural violence vary, though there is consensus that stress is multidimensional. Many studies, however, oversimplify this complexity by using cutoffs or cumulative indices that assign equal weight to stressors of differing severity and indiscriminately pool data across diverse background groups. Despite these limitations, research on Hispanics/Latinos offers key insights. Immigrant parents of Mexican heritage face workplace racial discrimination [14], acculturative stress [15], and family/economic strain [16], while Dominican and Mexican mothers report ethnic- and language-based discrimination [17]. Pre- and post-migration stressors—such as trauma, family separation, discrimination, and neighborhood disorder—often co-occur in Hispanic/Latino immigrant parents [11], revealing complex stress exposure.

Research indicates that various race-related stressors often intersect, yet studies rarely explore their combined impact on structural violence exposure among Hispanic/Latino parents [18]. Limited comparisons across immigrant and non-immigrant groups leave key gaps in understanding these stress patterns.

### Structural Violence-Induced Stress: Exploring Patterns by Nativity and Race/Ethnicity

Research on stress in Hispanic/Latino parents has been limited, often focusing on either immigrant parents or general groups without considering birthplace or time since migration. Studies comparing U.S.-born and foreign-born parents show that immigrant parents report higher levels of acculturative stress compared to their non-immigrant counterparts [19, 20]. Immigrants are also more likely to report financial strain, parenting stress, and racism. In contrast, non-immigrant parents report higher stress related to family dynamics, neighborhood issues, partner relationships, and life events [10]. Additionally, a broader study found that immigrant Hispanic/Latino adults experienced lower cumulative exposure to major traumatic events compared to non-immigrants [21]. While evidence suggests that stress exposure may vary by birthplace and time in the U.S., how stressors differ between foreign-born and U.S.-born Hispanic/Latino parents remains unclear.

### Present Study

Using data from the HCHS/SOL cohort, this study aimed to identify structural violence exposure patterns among Hispanic/Latino parents and examine how birthplace and years in the U.S. influence these patterns. We hypothesized a multi-class solution, including “*high-exposure*” and “*low-exposure*” groups, with foreign-born parents facing higher neighborhood disorder and acculturative stress, while U.S.-born parents would experience elevated chronic and perceived stress and racial discrimination [22].

## Methods

### Sample and Procedures

Details of HCHS/SOL, HCHS/SOL SCAS, and SOL Youth are published elsewhere [23–25]. HCHS/SOL is a U.S. population-based cohort study of 16,415 Hispanics/Latinos aged 18–74 years, selected through a two-stage probability sampling design from Chicago, Miami, Bronx, and San Diego [24].

The HCHS/SOL Sociocultural Ancillary Study (SCAS) enrolled 5,313 adult participants from the original HCHS/SOL cohort during the period of February 2010 and June 2011 [25]. SCAS participants returned within 9 months of their baseline exam to complete sociocultural and psychosocial measures. The SOL Youth ancillary enrolled the offspring of HCHS/SOL participants from the same four U.S. centers [23]. We retained 479 HCHS/SOL parents who participated in SCAS and provided SOL Youth offspring data. After excluding records with missing key variables, the final analytic sample consisted of 458 participants. All studies were approved by Institutional Review Boards at participating institutions.

### Study Measures

#### Sociodemographic Characteristics

Demographic factors from the HCHS/SOL baseline exam included age, sex, Hispanic/Latino heritage, household income, education, employment, marital status, and interview language (English vs. Spanish). Place of birth and years in the U.S. were categorized as: U.S.-born (limited to those born in the 50 states or Washington, D.C.), foreign-born residing in the U.S. for ≥ 10 years, and foreign-born residing in the U.S. for < 10 years.

#### Measuring Structural Violence Exposure: Multidimensional Stress Indicators

Six key indicators were used to assess structural violence exposure: (1) chronic stress, (2) perceived stress, (3) racial/ethnic discrimination, (4) intra-familial acculturative stress, (5) extra-familial acculturative stress, and (6) neighborhood disorder. This framework views stress exposure as structural violence, encompassing both overt racial stressors and subtle determinants tied to systemic inequities.

These six indicators were selected based on their established relationships with health disparities and their ability to capture various facets of structural violence, aligning with the World Health Organization’s Social Determinants of Health Framework, which emphasizes how social, economic, and political factors influence health outcomes and inequities [26–28]. Chronic and perceived stress capture overall stress burden [28, 29], while racial/ethnic discrimination reflects systemic racism, a core aspect of structural violence [30]. Acculturative stress (both intra- and extra-familial) addresses challenges unique to immigrant and minoritized populations [31], and neighborhood disorder represents the physical consequences of structural inequities [32]. Each indicator offers a distinct yet complementary perspective—cumulative exposure, subjective perception, race-based inequities, cultural tensions, and community-level factors—providing a comprehensive assessment of structural violence across multiple life domains [33]. Their inclusion in the LCA model is supported by prior literature and reinforced by our findings, which indicate modest to moderate correlations (*r* = 0.05–0.48) among domains. This range suggests related yet distinct constructs, each capturing unique dimensions of structural violence and contributing independently to the latent class structure [34].

#### Chronic Stress

Chronic stress was measured using the 8-item Chronic Stress Burden Scale [35], which assesses prolonged psychological stress across life domains. Respondents are asked about ongoing problems lasting six or more months in areas such as financial strain, employment difficulties, relationship issues, personal health concerns, health problems of close others, substance abuse in close others, caregiving responsibilities, and other chronic stressors. Each item was scored as “yes” or “no,” with affirmative responses summed to yield a total score ranging from 0 to 8. A median split distinguishing higher versus lower stress levels. The scale demonstrated acceptable internal consistency: α = 0.66 overall, 0.67 for the Spanish version, and 0.74 for the English version.

#### Perceived Stress

Perceived stress was measured using the 10-item Perceived Stress Scale (PSS-10; Cohen et al. [36]), which evaluates the degree to which individuals perceive their lives as unpredictable, uncontrollable, and overloaded over the past month. Each item is rated on a 5-point Likert scale, ranging from 0 (never) to 4 (very often), with higher scores indicating greater perceived stress. An example item includes, “In the last month, how often have you felt that you were unable to control the important things in your life?” Total scores were used to create a binary variable, with a median-split distinguishing high vs. low stress levels. Reliability estimates were α = 0.63, overall, 0.64 (Spanish), and 0.71 (English).

#### Racial/Ethnic Discrimination

Racial/ethnic discrimination was measured using the 17-item Brief Perceived Ethnic Discrimination Questionnaire-Community Version (PEDQ-CV; Brondolo, Kelly [37]), which evaluates individuals’ experiences of discrimination across various domains. The total score reflects exposure to exclusion/rejection, stigmatization/devaluation, work/school discrimination, and threat/aggression. A median-split was used to create a binary variable to distinguish high versus low levels of perceived discrimination. Reliability estimates were α = 0.63, overall, 0.63 (Spanish), and 0.75 (English).

#### Intra- and Extra-Familial Acculturative Stress

Acculturative stress was measured using the 17-item Hispanic Stress Inventory (HSI; Cervantes, Padilla [38]), comprising intrafamilial and extrafamilial subscales. Participants reported the occurrence and perceived stressfulness of events across various domains, including family conflicts, discrimination, and immigration-related challenges. An example item includes, “Because I am Latino I have had difficulty finding the type of work I want?” A median split distinguished high and low levels across domains. Both scales demonstrated acceptable internal consistency, with alphas ranging from 0.67 to 0.73 across language versions.

#### Neighborhood Disorder

Neighborhood disorder was measured using a 7-item scale from the Children of Immigrants Longitudinal Study [39], which evaluates residents’ perception of their neighborhood environment. Respondents rate the prevalence of vandalism, litter, and public disturbances. Higher scores indicate greater stress. Both continuous scores and a median split were used in the analyses. The scale showed moderate to strong internal consistency, per Cronbach’s alpha (overall: 0.74; Spanish: 0.75; English: 0.80).

### Analytic Strategy

Complex survey procedures were applied to account for sample weights and the two-stage sampling design, including clustering and stratification [24, 40]. Descriptive statistics summarize sample characteristics, stratified by place of birth and U.S. residency: U.S.-born (50 states/DC), foreign-born ≥ 10 years, and foreign-born < 10 years.

Latent profile analysis (LPA) was conducted to identify patterns of structural violence exposure, clustering individuals based on scores across continuous variables [41], using Mplus version 8.1. The optimal number of classes was determined through model fit indices: Lo-Mendall Rubin likelihood ratio test (LMR-LRT), Akaike Information Criteria (AIC), Bayesian Information Criteria (BIC), and sample size adjusted BIC, along with visual plots of estimated probabilities. Entropy was also considered for classification accuracy, with final class selection guided by model fit, theory, and interpretability [42].

Despite LPA findings indicating that a two-pattern solution for structural violence was not favored over a one-pattern model (LMR-LRT ≥ 0.05), we hypothesized the existence of distinct subgroups and, therefore, proceeded with latent class analysis (LCA) to capture potential heterogeneity in structural violence exposure. LCA has been widely applied in studies of social determinants and health disparities to identify unobserved subgroups, even when continuous indicators do not exhibit clear profile separation, as it allows for the classification of individuals based on shared exposure patterns rather than distributional assumptions alone [43]. LCA, utilizing the EM algorithm for full information maximum likelihood (FIML) estimation [44], is well-suited for categorical latent variables and allows for identifying unobserved cluster groups. Fit indices and theoretical considerations guided model selection. The Auxiliary (bch) function was employed to assess covariates, conducting pairwise chi-square tests to compare means across identified latent classes, enhancing the interpretability of emergent patterns.

We used a three-step multinomial logistic regression to examine structural violence patterns by place of birth and U.S. residency [45]. Place of birth and residency were dummy-coded (U.S.-born [reference], foreign-born ≥ 10 years, foreign-born < 10 years), with the *low-stress exposure* group as the reference. Models adjusted for age, sex, heritage, education, marital status, income, years in the U.S., employment, and interview language.

## Results

### Sample Characteristics

Table 1 presents participant characteristics stratified by place of birth and years in the U.S. The average age was 38.3 years of age (range: 23–71 years), with a majority being female (87.6% female). Most participants were of Mexican descent (45.9%) followed by Dominican (19.5%), Central/South American (12.4%), Cuban (11.1%), and Puerto Rican (9.8%). Low socioeconomic status was prevalent, with 32.7% reporting less than a high school education and 50.1% earning below $20,000 annually. The majority (84.9%) were foreign-born, with 72.2% residing in the U.S. for over 10 years. U.S.-born participants were generally younger, female, had higher socioeconomic status based on education and income, preferred English, and were more likely from the Bronx HCHS/SOL site.

**Table 1:** Baseline demographic characteristics for parents of SOL youth stratified by place of birth and length of stay in the U.S

Sample Characteristics	Total	U.S. Born	Foreign-born < 10 years	Foreign born ≥10 years	*p*-value
	*N* = 458	*n* = 69	*n* = 108	*n* = 281	
	µ (SE) or n (%)	µ (SE) or n (%)	µ (SE) or n (%)	µ (SE) or n (%)	
Age	38.27 (0.47)	35.78 (0.90)	37.07 (0.77)	39.57 (0.69)	< 0.01
Sex					
Male	57 (12.45)	2 (2.94)	16 (18.84)	39(12.05)	0.03
Female	401 (87.55)	67 (97.06)	92 (81.16)	242(87.95)	
Heritage Group					
Dominican	67 (19.47)	2 (4.02)	17 (19.06)	48 (24.08)	< 0.001
Central/South American	75 (12.35)	3 (4.92)	16 (12.56)	56 (14.38)	
Cuban	41 (11.10)	2 (3.93)	25 (24.25)	14 (6.73)	
Mexican	215 (45.93)	29 (49.50)	46 (39.38)	140 (48.10)	
Puerto Rican	52 (9.82)	29 (33.92)	4 (4.75)	19 (5.40)	
Mixed/Other	8 (1.33)	4 (3.70)	0 (0.0)	4 (1.30)	
Education					
Less than H.S.	154(32.72)	21(31.27)	28(22.72)	105(38.02)	0.01
H.S. or equivalent	134(30.43)	18(16.80)	39(39.78)	77(29.76)	
Greater than H.S.	170(36.85)	30(51.93)	41(37.50)	99(32.22)	
Marital Status					
Single	95(20.77)	21(29.03)	25(23.33)	49(17.16)	0.05
Married|Living w/ partner	287(65.48)	38(59.42)	77(70.66)	172(64.68)	
Separated|Divorced|Widow	76(13.75)	10(11.55)	6(6.01)	60(18.16)	
Household Income					
Less than $20k	228(50.13)	33(53.99)	56(53.62)	139(47.33)	0.56
$20k to $39,999k	144(31.20)	20(25.84)	31(25.59)	93(35.47)	
$40k and over	65(14.33)	16(20.17)	12(13.36)	37(13.14)	
Income missing	21(4.34)	0(.)	9(7.44)	12(4.06)	
Work Status					
Unemployed	245(57.11)	44(72.70)	62(53.66)	139(54.34)	0.06
Employed	213(42.89)	25(27.30)	46(46.34)	142(45.66)	
Language Preference					
Spanish	388(86.17)	21(38.17)	107(97.26)	260(94.48)	< 0.01
English	70(13.83)	48(61.83)	1(2.74)	21(5.52)	
Study Site					
Bronx	144(40.28)	28(39.89)	25(27.33)	91(46.73)	< 0.01
Chicago	91(12.58)	16(17.77)	11(8.81)	64(12.93)	
Miami	88(32.63)	1(2.13)	37(49.07)	50(31.51)	
San Diego	135(46.31)	24(68.30)	35(38.81)	76(44.22)	
Structural Violence Domains					
Chronic stress	1.88 (0.09)	0.82 (0.07)	2.43 (0.13)	3.45 (0.32)	< 0.01
Perceived stress	15.66 (0.47)	10.77 (0.46)	18.53 (0.63)	21.88 (1.00)	< 0.01
Ethnic discrimination	24.23 (0.54)	19.68 (0.28)	28.62 (0.90)	23.51 (0.93)	< 0.01
Intra-familial accult. stress	6.57 (0.55)	2.52 (0.34)	10.51 (0.97)	5.79 (1.39)	< 0.01
Extra-familial accult. stress	11.24 (0.78)	5.37 (0.95)	18.85 (1.11)	2.98 (0.58)	< 0.01
Neighborhood disorder	7.95 (0.19)	7.11 (0.19)	8.13 (0.34)	10.16 (0.50)	< 0.01

### Optimal Latent Class Configuration

Fit indices suggested the best-fitting model was either a three- or four-class solution, with the lowest AIC, BIC, and aBIC values (Table 2). AIC and aBIC favored a four-class solution (3557.72 and 3583.46), while BIC supported a three-class solution (3656.70). Entropy increased with additional classes, exceeding 0.80 for the five- and six-class solutions, but these led to very small clusters (as few as 13 observations) and offered minimal improvement in entropy. Log-likelihood and LMR-LRT results were inconclusive.

**Table 2:** Model fit indices for latent class analysis with HCHS/SOL sampling weights (*n* = 458)

No. of classes	-2LL	AIC	BIC	aBIC	Entropy	Smallest Class	LMR-LRT *p*-value
Latent Class Analysis (Categorical Indicators)							
1	-1895.867	3803.735	3828.496	3809.454	N/A	N/A	N/A
2	-1786.446	3598.892	3652.541	3611.283	0.66	183	0.109
3	-1767.079	3574.159	**3656.696**	3593.222	0.74	40	0.578
4	-1751.862	**3557.724**	3669.149	**3583.459**	0.79	61	0.419
5	-1747.248	3562.497	3702.810	3594.904	0.81	13	0.633
6	-1743.704	3569.408	3738.610	3608.488	0.83	13	0.522

Note: -2LL = negative 2 log likelihood; AIC = Akaike Information Criteria; BIC = Bayesian Information Criteria; aBIC = sample size adjusted Bayesian Information Criteria; LMR = Lo-Mendell-Rubin adjusted likelihood ratio test; BLRT = bootstrapped likelihood ratio test. The best fitting indices are in bold

We plotted two-, three-, and four-class solutions to finalize the model. In the two-class solution, 62.2% had high structural violence exposure and 37.8% had low exposure. Sensitivity analyses showed similar results using continuous domain scores. The three-class solution included a *low-exposure* group (41.3%), a *high-exposure* group (47.8%), and a *high-environmental exposure* group (10.9%). Unlike the *high-exposure* group, which experienced consistently high levels across all six indicators, the *high-environmental* exposure group exhibited a more localized stress pattern, with elevated environmental (e.g., neighborhood disorder) and psychological stressors but relatively lower exposure to discrimination and acculturative stress. Although the *high-environmental* exposure group was relatively smaller, its distinct neighborhood disorder and psychological stress pattern warranted retention. Model selection confirmed that the three-class structure best captured the heterogeneity in structural violence exposure.

**Fig. 1:**
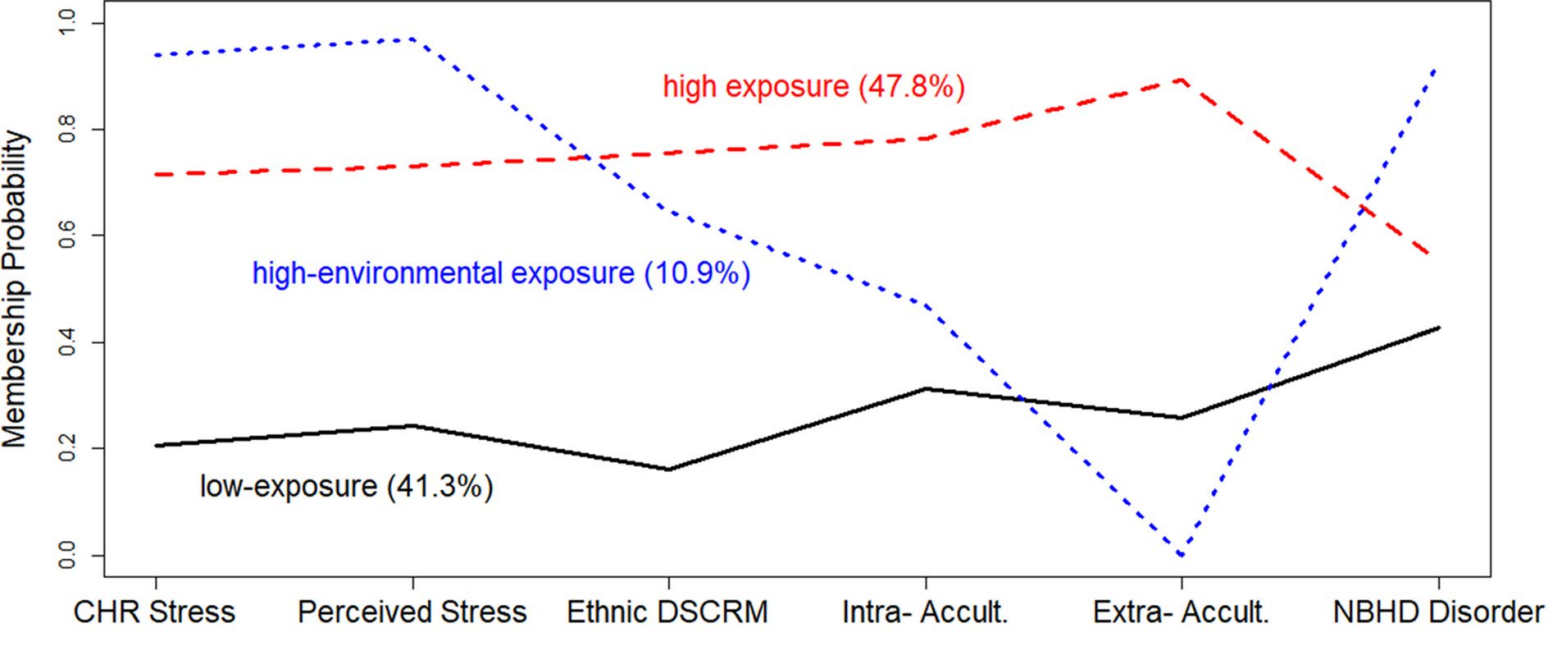
Probability membership plot for 3-class solution using Latent Class Analysis (LCA). Black solid line: low-exposure(41.3%); Red dashed line: high-exposure (47.8%); Blue dotted line: high-environmental exposure (10.9%). CHR Stress= Chronic stress. Ethnic DSCRM= Ethnic discrimination. Intra- Accult = Intra-familial acculturative stress. Extra- Accult = Extra-familal acculturative stress. NBHD Disorder = Neighborhood disorder

The four-class solution resembled the three-class, differing mainly in neighborhood disorder exposure. Although the four-class solution slightly improved model fit indices, it primarily subdivided the high-environmental exposure group without providing additional conceptual clarity. Given our goal of identifying distinct and interpretable structural violence exposure patterns, the three-class solution was deemed most appropriate. Additionally, while the *high-environmental* exposure group comprised 10.9% of the sample, it retained a meaningful and theoretically relevant profile, justifying its inclusion [46, 47]. Thus, we chose the more parsimonious three-class model, which best captured the sample’s heterogeneity (Fig. 1).

### Characterization of Latent Class Profiles

Table 3 presents demographic characteristics by structural violence patterns. *The high-environmental* exposure group had a higher proportion of females (98.6%) compared to the *low-exposure* (83.4%) and *high-exposure* (88.6%) groups. In the *low-exposure* group, 53.1% reported household incomes over $20,000, which was higher than in the *high-exposure* group, though income did not differ significantly for the *high-environmental* exposure group. Marriage rates were higher in the *high-environmental* exposure group (86.3%) than in the *low-* (65.8%) and *high-exposure* (60.4%) groups, while employment was lowest in the *high-environmental* group (9%). Additionally, more participants in the *low-* and *high-exposure* groups were foreign-born with less than 10 years in the U.S.

**Table 3:** Demographic characteristics by latent class membership

	Latent cluster 1: Low-exposure	Latent cluster 2: High-exposure	Latent cluster 3: High-environmental exposure	Pairwise Significant Difference (*p* ≤ 0.05)
	*n* = 208 41.3%	*n* = 210 47.8%	*n* = 40 10.9%	
Demographic Characteristics				
Age	38.6	37.9	38.8	NS
Sex				
Male	16.6%	11.4%	1.4%	C1 > C3; C2 > C3
Female	83.4%	88.6%	98.6%	C3 > C1; C3 > C2
Hispanic/Latino heritage group				
Puerto Rican	6.4%	10.3%	20.5%	NS
Cuban	13.3%	11.2%	2.3%	C1 > C3
Dominican	21.5%	12.6%	41.7%	NS
Central/South American	14.4%	12.4%	4.6%	NS
Mixed/Other	1.8%	1.3%	0.0%	NS
Mexican American	42.6%	52.2%	31.3%	NS
Educational attainment				
Greater than H.S.	28.8%	38.5%	22.2%	NS
H.S. or equivalent	30.7%	26.9%	44.8%	NS
Less than H.S.	40.5%	34.6%	33.0%	NS
Annual household income				
≥$20K	53.1%	34.1%	66.9%	C1 > C2
Less than $20K	40.7%	61.9%	34.3%	C2 > C1
Income missing	6.2%	4.0%	0.0%	C1 > C3; C2 > C3
Marital status				
Single	19.4%	24.2%	11.0%	NS
Married/Living with a partner	65.8%	60.4%	86.3%	C3 > C1; C3 > C2
Separated/Divorced/Widowed	14.7%	15.4%	2.7%	C2 > C3
Work status				
Employed	52.7%	42.2%	9.0%	C1 > C3; C2 > C3
Unemployed	47.3%	57.8%	91.0%	C3 > C1; C3 > C2
Language preference				
English	12.8%	12.1%	25.5%	NS
Spanish	87.2%	87.9%	74.5%	NS
Nativity Status				
U.S.-Born	10.1%	14.3%	46.8%	NS
Foreign-born < 10 years	33.9%	29.3%	0.0%	C1 > C3; C2 > C3
Foreign-born ≥ 10 years	56.1%	56.4%	57.3%	NS

Table 4 shows multinomial logistic models examining the relationship between covariates and structural violence exposure clusters. Significant associations were found for Hispanic/Latino heritage and income. Puerto Ricans had higher odds of being in the *high-environmental exposure* group compared to the *low-exposure* group (OR: 5.63, 95% CI: 1.17, 27.16), while Dominicans had lower odds of being in the *high-exposure* group compared to the *low-exposure* (OR: 0.35, 95% CI: 0.14, 0.85). Additionally, those with annual incomes over $20,000 were less likely to be in the *high-exposure* group than the *low-exposure* group.

**Table 4:** Results from multinomial logistic regression models evaluating the association of covariates and structural violence cluster membership

	High-exposure vs. Low-exposure*		High-environmental exposure vs. Low Exposure*	
**Covariate**	**Odds ratio** **(95% CI)**	***p*** **-value**	**Odds ratio** **(95% CI)**	***p*** **-value**
Age	1.03 (0.99, 1.07)	0.22	1.00 (0.92, 1.08)	0.90
Sex				
Male	0.77 (0.36, 1.68)	0.52	1.68 (0.08, 34.61)	0.74
Female	Ref	1.0	Ref	1.0
Hispanic/Latino heritage group				
Puerto Rican	0.91 (0.28, 2.99)	0.88	**5.63 (1.17**,** 27.16)**	**0.03**
Cuban	1.04 (0.41, 2.65)	0.94	2.06 (0.14, 78.00)	0.60
Dominican	**0.35 (0.14**,** 0.85)**	**0.02**	5.11 (0.64, 40.68)	0.12
Central/South American	1.08 (0.53, 2.18)	0.84	2.41 (0.35, 16.65)	0.37
Mixed/Other	0.62 (0.08, 5.10)	0.66	**0.00 (0.00**,** 0.00)**	**0.00**
Mexican American	Ref	1.0	Ref	1.0
Educational attainment				
Greater than H.S.	1.42 (0.74, 2.74)	0.29	0.74 (0.09, 6.03)	0.78
H.S. or equivalent	1.23 (0.65, 2.33)	0.53	1.00 (0.27, 3.71)	0.99
Less than H.S.	Ref	1.0	Ref	1.0
Annual household income				
>$20K	**0.54 (0.30**,** 0.95)**	**0.03**	1.04 (0.28, 3.84)	0.95
Income missing	0.54 (0.18, 1.66)	0.28	**0.00 (0.00**,** 0.00)**	**0.00**
Less than $20K	Ref	1.0	Ref	1.0
Marital status				
Single	1.00 (0.42, 2.38)	0.99	1.62 (0.12, 21.25)	0.71
Married/Living with a partner	0.61 (0.29, 1.28)	0.19	3.47 (0.30, 40.01)	0.32
Separated/Divorced/Widowed	Ref	1.0	Ref	1.0
Work status				
Employed	0.69 (0.41, 1.19)	0.18	0.43 (0.13, 1.37)	0.15
Unemployed	Ref	1.0	Ref	1.0
Language preference				
English	0.52 (0.17, 1.63)	0.27	1.33 (0.02, 115.8)	0.90
Spanish	Ref	1.0	Ref	1.0

* Models adjusted for place of birth and U.S. length of residence

Statistically significant at *p* < 0.05 (bolded values)

### Multinominal Regression of Birthplace and U.S. Residency on Impacts of Structural Violence

Multinomial logistic regression models assessed the association of place of birth and years in the U.S. with structural violence group membership, using the *low-exposure* group as the reference (Table 5). Neither place of birth nor years in the U.S. significantly predicted membership in the *high-exposure* group compared to *low-exposure* (OR: 0.49, 95% CI: 0.16–1.51; OR: 0.73, 95% CI: 0.26–2.06, respectively). However, foreign-born adults residing in the U.S. for less than 10 years had an extremely low likelihood of being in the high-environmental exposure group compared to U.S.-born adults (OR: <0.01, 95% CI: <0.01–<0.01), even after adjusting for covariates of age, sex, Hispanic/Latino background, educational attainment, marital status, annual income, employment status, and interview language Sensitivity analyses showed no significant difference when nativity was dichotomized (*p* = 0.10, *p* = 0.26, respectively).

**Table 5:** Logistic regression models for the association of place of birth and length of U.S. Residence with latent clusters of structural violence, adjusting for covariates.^a^

	High-exposure vs. Low-exposure		High Socio-environmental vs. Low-exposure	
Place of Birth and U.S. Length of Residence	OR (95% CI)	*p*-value	OR (95% CI)	*p*-value
U.S.-born	Ref		Ref	
Foreign-born with Less than 10 years	0.49 (0.16, 1.51)	0.21	**0.0 (0.00**,** 0.00)**	**0.00**
Foreign-born with 10 years or more	0.73 (0.26, 2.06)	0.55	0.37 (0.0, 119.6)	0.73

^a^Adjusted for age, sex, Hispanic/Latino heritage group, education, marital status, annual household income, employment status, and language of interview (English or Spanish)

Statistically significant at *p* < 0.05 (bolded values)

## Discussion

Our analysis uncovered three distinct patterns of structural violence exposure among Hispanic/Latino parents: *high-exposure*, *low-exposure*, and *high-environmental exposure* categories. Notably, nearly half of the participants (47.8%) fell into the *high-exposure* group, highlighting the widespread prevalence of chronic stress, ethnic discrimination, acculturative stress, and neighborhood disorder in this population. In contrast, the *high-environmental* exposure group (10.9%) was characterized by elevated neighborhood disorder and psychological stress but relatively lower discrimination and acculturative stress levels. These distinctions are important, as they suggest that while some individuals experience a broad range of structural violence stressors, others are primarily affected by their immediate social and physical environments. Recognizing these variations is critical for developing targeted interventions that address systemic inequities and localized structural conditions.

Our findings are aligned with prior research showing that Hispanic/Latino adults, particularly those born in the U.S., experience high levels of structural violence [48]. Evidence indicates that Hispanic/Latino parents experience higher levels of parenting stress and everyday racism than non-Hispanic White parents [10], alongside workplace discrimination [49] and stress related to economic hardships and immigration challenges [50]. Our study emphasizes the need to explore factors contributing to structural violence within this population. Indeed, structural violence in Hispanic/Latino communities is multidimensional, with subgroup differences shaped by nativity, socioeconomic status, and social environment.

Place of birth and length of U.S. residency were key factors in structural violence group membership. Foreign-born Hispanic/Latino parents with less than 10 years in the U.S. were less likely to fall into the *high-environmental exposure* category compared to their U.S.-born counterparts. Despite the challenges of migration, research suggests that foreign-born individuals often display greater optimism, resilience, and fortitude [51, 52]. This may reflect the “healthy immigrant effect,” where migrants arrive with the health and resources to endure the migration process [53], potentially shaping their perceptions of structural violence [54]. Perceptions of structural violence among immigrants may also be influenced by factors such as the pursuit of social mobility, which can positively shape their view of their new environment [55]. Additionally, foreign-born individuals often benefit from robust, more cohesive social networks that provide greater support and social capital [56]. As migration patterns shift and U.S.-born populations grow, understandings of structural violence exposure are likely to evolve. Indeed, as economic hardships persist and exposure to structural inequalities accumulates over time, stress profiles may shift, aligning more closely with those of U.S.-born individuals.

Our findings reinforce the need for policy and community-level interventions tailored to different groups’ unique stressors. Demographic factors shaped structural violence exposure, with Puerto Rican individuals and those with lower household incomes experiencing higher exposure levels. This finding aligns with research linking poverty to increased stress, psychological distress, and neighborhood disorder [57]. Thus, while broad structural reforms remain necessary, programs targeting neighborhood-level conditions—such as housing stability, economic investment, and reducing environmental stressors—may be particularly relevant for individuals in the *high-environmental* exposure group [58]. Conversely, interventions addressing chronic stress, discrimination, and acculturative stress should prioritize those in the *high-exposure* group, who face a more comprehensive burden of structural violence-related stressors. Further research should examine whether structural violence exposure varies by country of origin or settlement region. In our study, Puerto Ricans, primarily recruited from the Bronx—where nearly 30% of residents live below the poverty line—faced concentrated socioeconomic disadvantages. Considering these contextual factors is essential for designing targeted interventions.

Limitations should be considered when interpreting this study’s findings. First, structural violence profiles relied on self-reported data, which may introduce response bias. Although using median splits may have constrained exposure measurement, treating exposure variables as continuous in latent profile analyses showed similar patterns. Objective measures (e.g., cortisol) and additional stress-related structural violence subtypes were not included. While the structural violence indicators we used are interrelated, each represents a distinct construct capturing unique dimensions, though some conceptual overlap is inherent in complex social exposures. Second, low entropy values, likely due to small cell sizes, limited cluster assignment strength. Although the *high-environmental* exposure group is small, its distinct stress pattern warranted retention based on model fit indices and theoretical relevance. While small subgroups are a limitation in latent profile analyses, prior research supports their inclusion when they capture meaningful variations [46, 47, 59]. Future studies should confirm these findings with larger samples. Third, generalizability may be restricted, as recruitment was confined to four large urban cities. While site-specific analyses were not conducted, this study was designed to capture overarching patterns of structural violence rather than regional policy effects. Future research should examine how state, county, and city-level policies and legal frameworks contribute to these disparities, as structural violence may manifest differently across geographic and sociopolitical contexts.

Beyond these considerations, grouping Central and South American individuals may mask subgroup differences, underscoring the need for future research examining heterogeneity within Hispanic/Latino populations, including those of mixed origins. This study did not account for diversity in sexual orientation, an important factor that may intersect with structural violence experiences and should be explored in future work. With 87.6% female respondents, these findings likely reflect the experience of mothers and may not fully generalize to more gender-diverse populations. Future research should examine whether similar structural violence patterns emerge in more gender-balanced samples. Finally, given that U.S.-born Hispanic/Latino parents comprised a smaller portion of the sample, findings related to this group should be interpreted with caution, as they may not fully capture the range of structural violence experiences among non-immigrant Hispanic/Latino caregivers.

These findings lay a critical foundation for understanding the varied forms of structural violence and race-related stress experienced by Hispanic/Latino parents. By identifying groups most vulnerable to specific stressors, this knowledge supports the development of targeted preventive interventions. For example, addressing high stress exposure and neighborhood disorder among U.S.-born Hispanics/Latinos is an urgent public health priority. Future research should examine whether high exposure to structural violence leads to adverse health outcomes over time and if these effects impact future generations, shaping the health and well-being of their children.

## Data Availability

Data Availability Data access may be granted or denied at the discretion of the principal investigator. If granted, it will require a data-use agreement and undergo a rigorous review process to assess the purpose and research aims.Code Availability Access to the analysis code may be granted at the discretion of the principal investigator, pending review and if deemed reasonable.
